# Formation Mechanism of Skin-Core Chemical Structure within Stabilized Polyacrylonitrile Monofilaments

**DOI:** 10.1186/s11671-019-2926-x

**Published:** 2019-03-13

**Authors:** Yang Sha, Wei Liu, Yue Li, Weiyu Cao

**Affiliations:** 1 State Key Laboratory of Organic-Inorganic Composites, Beijing University of Chemical Technology, Beijing, 100029 China; 2 The Key Laboratory of Education Ministry on Carbon Fiber and Functional Polymer, Beijing University of Chemical Technology, Beijing, 100029 China

**Keywords:** Skin-core structure, Stabilized PAN fiber, Photo-induced force microscopy

## Abstract

Although it has been half a century since polyacrylonitrile (PAN)-based carbon fibers were first developed, the exact formation mechanism of skin-core structure of PAN-based carbon fibers, especially the stabilized PAN fibers, was still not well clarified from the viewpoint of the chemical structure. In order to address this aforementioned challenge, a powerful tool with nanoscale resolution named photo-induced force microscopy was applied to map the chemical group distribution in the cross section of stabilized PAN fibers and reveal the evolution mechanism of skin-core structure throughout the whole stabilization process. The results indicated that the formation of skin-core structure of stabilized PAN fiber was attributed to the complex and overlapped chemical reactions caused by gradient of oxygen along radial direction and the formation of dense crystal layer at the interface between the skin and core part. Finally, the crystal layer was destroyed and the monofilaments tended to be homogeneous with further oxidation.

## Introduction

PAN-based carbon fiber (CF) is a frontier material with high tensile strength and Young’s modulus, as well as excellent heat resistance. Due to its superior properties, it has been applied broadly as the reinforced structural material in aviation, aerospace, and other new industrial fields [1–3]. Currently, the strongest commercially available carbon fibers possess tensile strength of ~ 7 GPa. However, based on –C–C bond strength calculations with ideal graphite model, the theoretical strength of carbon fibers is around 180 GPa [4]. The enormous gap between real and theoretical tensile strength is mainly attributed to the heterogeneous skin-core structure of carbon fiber. This structural heterogeneity results in uneven stress distribution within carbon fiber monofilament. Destruction tends to happen in the area, which suffers higher stress, thus leading to the breakage of carbon fiber [5–7]. Hence, it is of great significance to figure out the formation mechanism of this structural defect and minimize its effect on the properties of the resultant carbon fibers.

The manufacture of carbon fibers involves three steps including spinning of PAN precursors, thermal stabilization, and carbonization. Among these, thermal stabilization is the most complex step which involves reactions such as cyclization, dehydrogenation, and oxidation. Cyclization reaction leads to the generation of cyclized structures and the conversion from –C≡N to –C=N. Dehydrogenation reaction is associated with the formation of –C=C. Carbonyl groups are introduced after the precursor fibers undergo oxidation reaction [2, 8]. The stabilization process contributes to the transformation from linear PAN chains to infusible and heat-resistance ladder structure, which is necessary for the carbonization process [9–11]. The preparation of PAN-based carbon fiber is a continuous process, to put it another way, and the final heterogeneous skin-core structure in carbon fiber is mainly inherited from the stabilized PAN fiber. Therefore, revealing the formation mechanism of skin-core structure of stabilized PAN monofilament especially the chemical structural distribution is beneficial to minimize the structural heterogeneity within carbon fiber.

There have been numerous studies focused on the stabilization of PAN fibers. However, the investigations about skin-core structure of stabilized PAN fibers are very limited. Lv et al. [12] reported that the heterogeneous oxygen diffusion from skin to core results in the formation of a dense skin region, which retards further diffusion of oxygen and leads to the formation of skin-core structure. Nunna et al. [13] used Raman spectroscopy and elemental analysis to reveal skin-core structure of stabilized fibers. These elegant works have contributed greatly to the study of skin-core structure of the stabilized PAN fibers. However, they mainly focus on the radial mechanical property of stabilization PAN fibers rather than chemical structure, the detailed structural information is still not very clear. Hence, the equipment with high spatial resolution is necessary for the study of the skin-core chemical structure of stabilized PAN fibers at different stages of stabilization process.

**Fig. 1:**
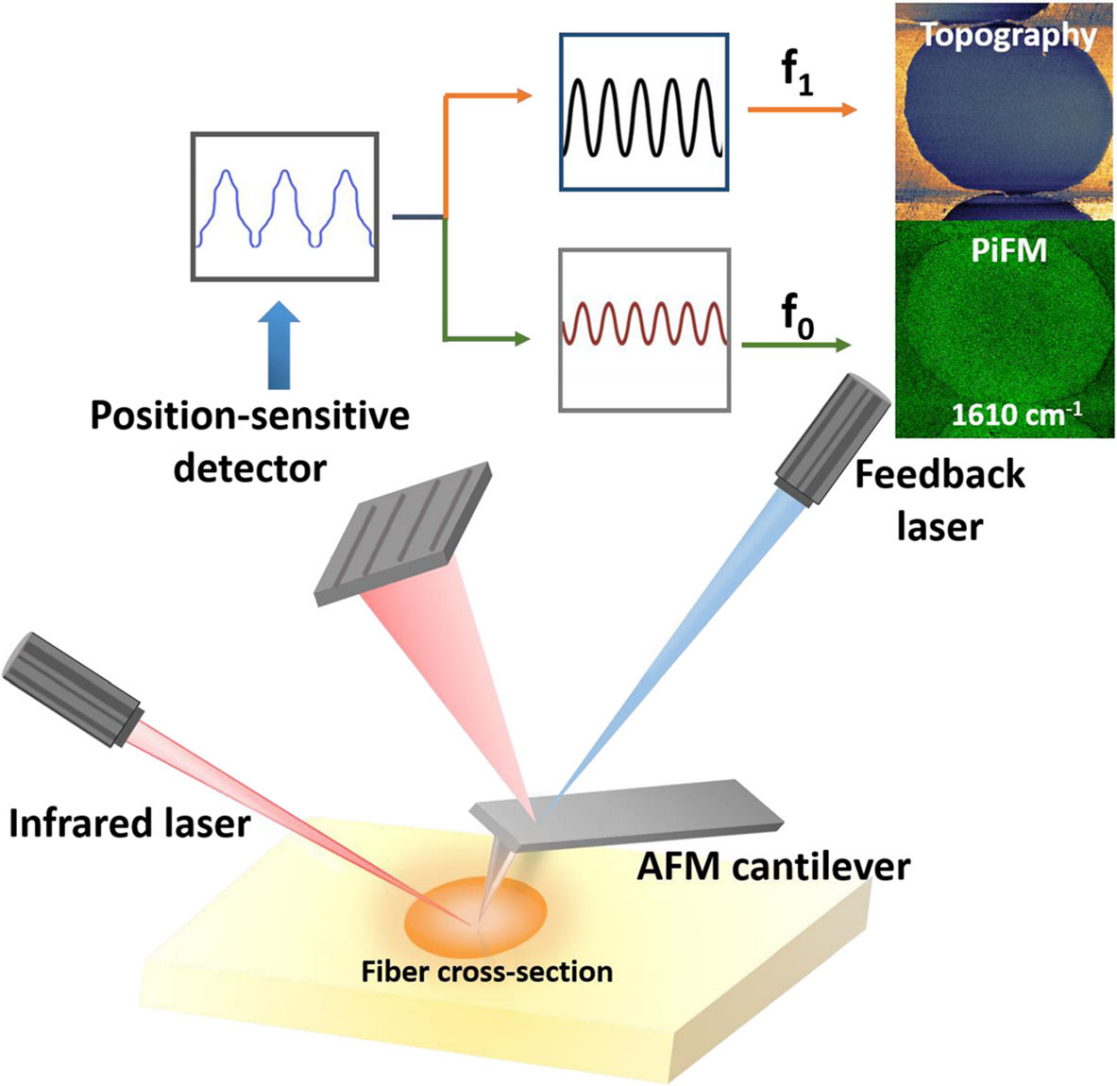
Simplified schematic of the photo-induced force microscopy (PiFM) setup

In this study, photo-induced force microscopy (PiFM) was applied to analyze the formation mechanism of skin-core chemical structure within stabilized PAN monofilaments at different temperatures. As shown in Fig. 1, PiFM is a cutting-edge scanning probe microscopy technique that combines an atomic force microscopy (AFM) tip with a tunable infrared laser to induce a dipole for chemical imaging. It can offer a lateral resolution of ~ 10 nm. There is a pulse at *f*_*m*_ = *f*_1_ − *f*_0_, where *f*_0_ and *f*_1_ are the first and second mechanical eigenmode resonances of cantilever. Topography and PiFM signal of the sample are simultaneously recorded by the AFM feedback system at *f*_1_ and *f*_0_, respectively [14].

## Methods

### Sample Preparation

Samples from different stages of stabilization under different ambient temperatures were collected. The PAN fibers used in this study are the 6 K precursor fibers of HENGSHEN T700 (HENGSHEN Co. Jiangsu, CHINA). The precursor fibers were continuously passing through five ovens with gradually increasing temperatures (210 °C, 220 °C, 230 °C, 240 °C, 250 °C). The samples were denoted to 01–05 sequentially. Stabilization time in each oven was 8 min, and the running speed of the tow was 30 m/h.

The procedure for preparing samples for PiFM measurements is as follows: Firstly, a fiber tow is attached straightly on the bottom of the model to ensure that the fiber axis is parallel and close to the epoxy block surface and then embedded in epoxy resin. To acquire the transverse section, the surface perpendicular to the fiber axis was mechanically grinded and polished by a polishing machine (Struers Inc.).

### Characterization

PiFM (Molcular Vista, USA) measurements were performed to investigate the changes of functional groups in different radial positions of the monofilament during the stabilization and were operated in non-contact to prevent the softest samples from damage and achieve higher spatial resolution than AFM topography.

Raman spectroscopy was performed with a × 100 objective by using the 532-nm laser of confocal Raman spectroscopy (RM2000, Renishaw, UK).

## Results and Discussions

**Fig. 2:**
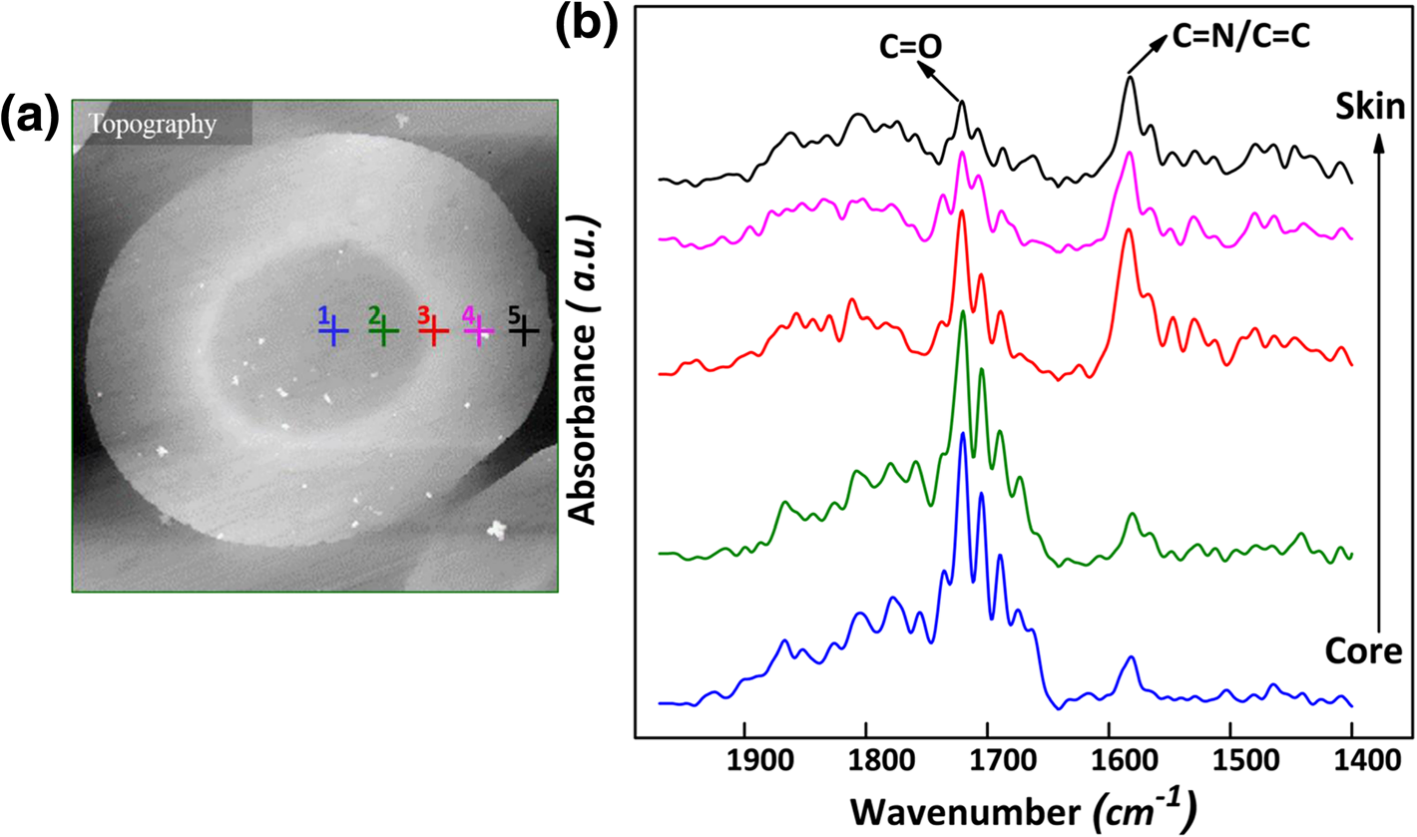
**a** Topography image of sample 03; **b** The spectra in 1400–1900 cm^−1^of different points along radial direction

Figure 2b presents the typical PiFM spectra in 1400–1900cm^−1^ region at different positions along radial direction. The absorption band around 1580 cm^−1^is due to combination vibrations of –C=C and –C=N stretching modes [15]. The absorption band around 1720 cm^−1^is assigned to the *ν*_C=O_. It could be observed that the intensity of these two bands changed with positions. This phenomenon was owing to the various chemical structures formed by the different reactions along radial direction during stabilization. However, the evolution of chemical skin-core structure in the monofilaments could not be revealed visually. Hence, PiFM mapping was carried out at both vibration modes with nanoscale specificity.

**Fig. 3:**
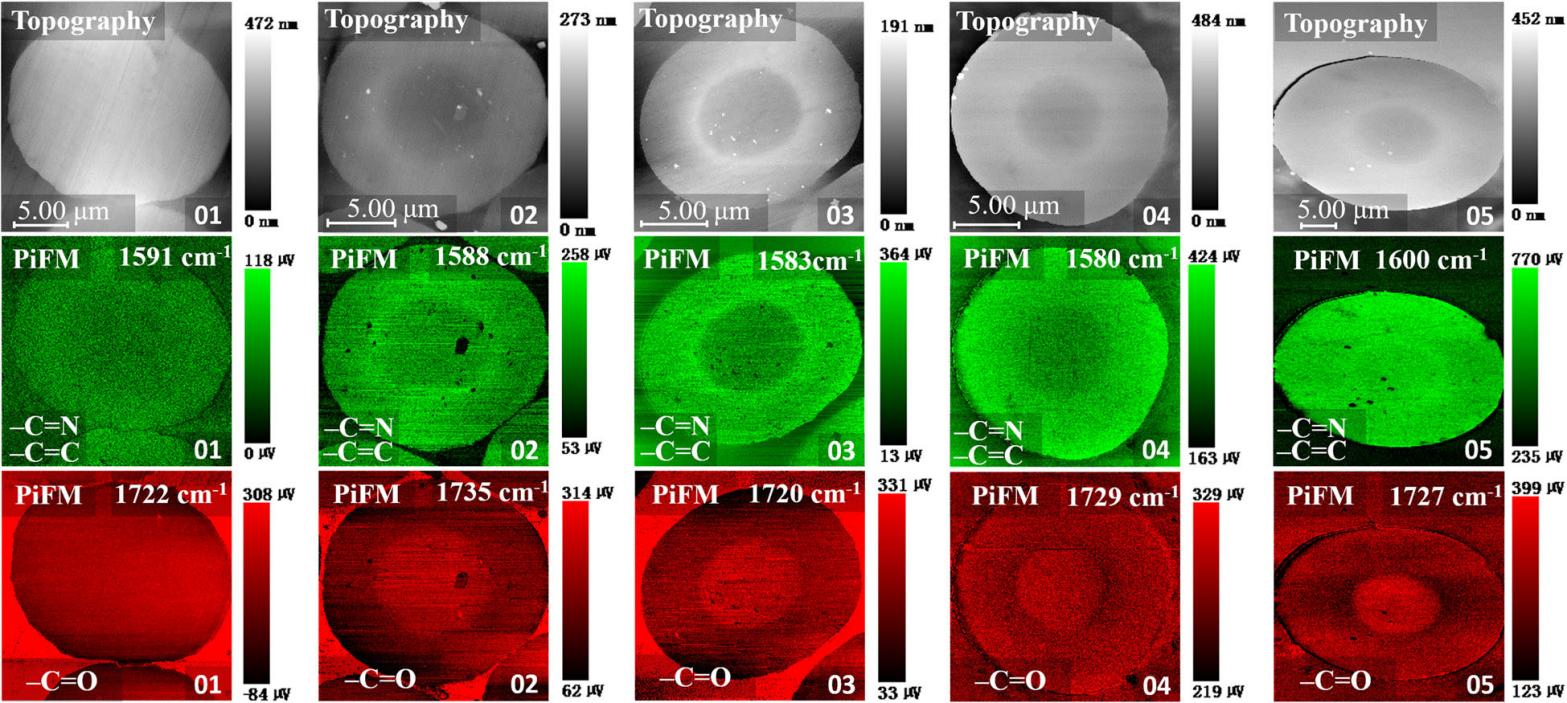
Topography of samples 01–05; PiFM mapping of absorbance intensity at 1600 and 1730 ${\mathsf{cm}}^{-\mathsf{1}}$ for samples 01–05

**Scheme 1:**
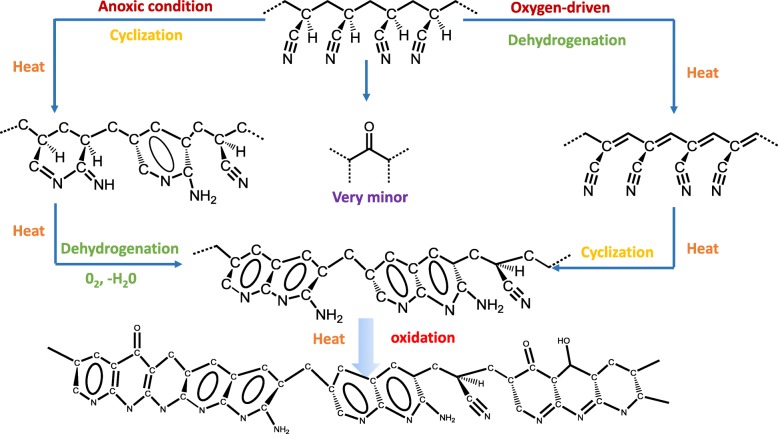
Proposed structural changes during stabilization

Figure 3 shows the topography and the PiFM mapping of absorbance intensity at 1600 and 1730 cm^−1^of samples 01–05. The intensity of *ν*_–C=C_ and *ν*_–C=N_ at 1600 cm^−1^of the core was obviously smaller than that of the skin. This was ascribed to the different chemical reactions caused by the oxygen concentration gradient distribution in the cross section. Proposed chemical reaction schemes of heat-treated PAN are illustrated in Scheme 1, the dehydrogenation is mainly driven by oxygen, while anoxic condition is more conductive to the occurrence of cyclization [16]. At the initial stage, more oxygen was concentrated in the skin part, so this part tended to happen by dehydrogenation reaction and generated more unsaturated bonds. The unsaturated bonds formed in the skin part enhanced the overall intensity at 1600 cm^−1^. Furthermore, there was a bright ring appearing at the interface between the skin and the core of samples 02 and 03, which can be ascribed to the formation of crystal layer at the interface. Nunna et al. [17] have proved that the mechanical properties of the skin and core are different, and the reduced modulus of the skin is higher than the core. Although the skin and core experienced a same strain as a function of stretching force during stabilization, the deformation resistance ability of molecular chains in the skin was higher than the core because of a higher modulus. Therefore, there was a shearing force emerging at the interface between the skin and core part. In this case, molecular chains in interface region would stack more efficiently and regularly under shearing force, thus generating a higher density of functional group –C=N and –C=C. According to the Lambert-Beer law, there should be an enhancement of the infrared absorption intensity, resulting in the appearance of the bright ring. In addition, the thin and dense crystal layer further retarded the diffusion of oxygen towards the core. Therefore, the skin-core difference of sample 03 was further enlarged. However, as the stabilization process went on, the bright ring gradually disappeared and the monofilaments tended to be homogenous as shown in Fig. 3 04–05. It was because that the further oxidation leads to the destruction of crystal barrier layer, which was beneficial to further oxygen diffusion and dehydrogenation in the core part. This is also in good accordance with the phenomenon that the crystallinity of stabilized PAN fiber presents increasing initially, then shows a continuous decrease with the increasing of temperature [18].

On the other hand, although the overall intensity at 1730 cm^−1^ showed almost no increase until sample 04, obvious skin-core difference was observed in samples 02 and 03. This is because the PAN was obtained by the copolymerization of acrylonitrile and itaconic acid which contains carbonyl group. At the initial stage, dehydrogenation reaction tended to happen in the skin part, so carbonyl group was eliminated in the form of H_2_O. Therefore, the core part has a higher concentration of carbonyl group. With the further stabilization, the higher temperature and the improved uniformity of oxygen content along radial direction promoted oxidation in the skin and dehydrogenation in the core simultaneously in samples 04 and 05. The oxidation not only involved the formation of –C=O bonds, but also enhanced the dehydrogenation by eliminating hydrogen in the form of H_2_O [19]. As shown in Fig. 3, it is clear to observe that the conjugated and the oxidized structures tend to be homogenous in the samples 04 and 05 in terms of the absorbance intensities at 1600 and 1730 cm^−1^.

**Fig. 4:**
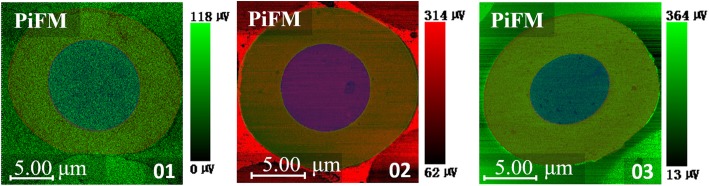
Micro-area analysis on the image

As seen in Fig. 3, the samples were mainly rich in –C=O in the core region and rich in –C=N/−C=C in the skin region. Figure 4 shows PiFM mapping of samples 01–03. For quantification, the ratio of I_–C=O_/I_–C=N/−C=C_ was calculated and shown in Table 1, which was considered as the ratio of oxidized structure to conjugated structure. There was a clear decrease from samples 01 to 03, showing that the higher concentration of carbon-carbon double bonds was generated after further dehydrogenation reaction in the skin region.

**Table 1:** The ratio of I_–C=O_/I_–C=N/−C=C_ from samples 01–03

Samples	01	02	03
I_1730 cm_^−1^/I_1600 cm_^−1^	0.48	0.37	0.27

**Fig. 5:**
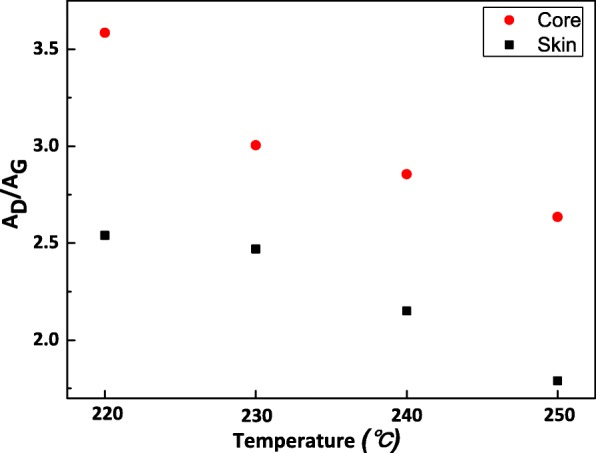
The *A*_D_/*A*_G_ value in skin and core regions of fibers with respect to treatment temperature from 220 °C to 250 °C

Raman measurements for the cross section of fibers were performed to further prove that dehydrogenation reaction domains in the skin region. The integral area ratio of D to G band *A*_D_/*A*_G_ value is considered as the sp^2^/sp^3^–C ratio [20]. Figure 5 shows the *A*_D_/*A*_G_ value in the skin and core regions of fibers with respect to treatment temperature from 220 °C to 250 °C (There was almost no D and G band signal of sample 01, which is due to low degree of dehydrogenation reaction and strong fluorescence effect caused by organic substance). A significant difference existed between the skin and core, the skin part having higher concentration of sp^2^ hybrid carbon atoms. This was attributed to higher degree of dehydrogenation reaction in the skin part, which leads to the formation of –C=C. As the stabilization process went on, the *A*_D_/*A*_G_ value slightly decreased, indicating the higher degree of graphitization. This is in good accordance with PiFM mapping results.

**Fig. 6:**
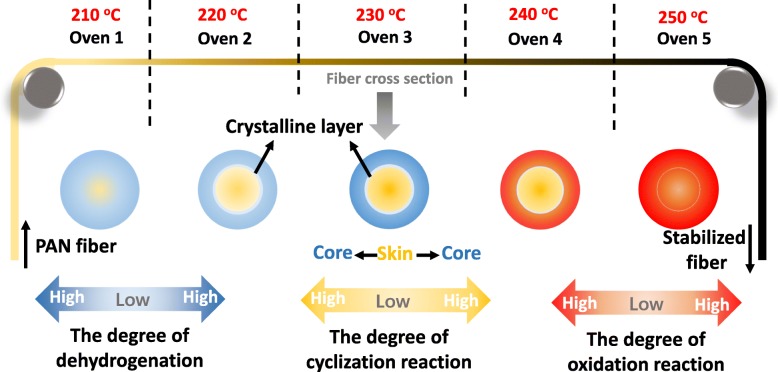
Formation mechanism of the skin-core structure of stabilized PAN fibers

In order to describe the formation of skin-core chemical structure of PAN-stabilized fiber schematically, an overall diagram of the most probably formation mechanism is drawn in Fig. 6. The different reactions are labeled by corresponding color, blue represents dehydrogenation, yellow represents cyclization, and red labels the oxidation. The formation of the skin-core chemical structure was caused by cyclization domain in core region while the skin part underwent the oxygen-driven dehydrogenation domain. This can be attributed to the heterogeneous oxygen distribution in the skin and core part. Besides, the structural heterogeneity was also increased by the crystal layer formed at the interface between the skin and core. With the stabilization process developing, the crystal layer was destroyed by oxidation. Sequentially, the increased extent of oxidation within the whole monofilament could promote the fiber to be homogenous apparently.

## Conclusions

This study shows that the skin-core structure of stabilized PAN fibers initially formed by cyclization occurred in a core region while the skin part underwent the oxygen-driven dehydrogenation domain. Then, with the higher degree of oxidation, the filaments could tend to be homogenous.

## Abbreviations

AFM: Atomic force microscopy
PAN: Polyacrylonitrile
PiFM: Photo-induced force microscopy
